# Clonal diversity impacts coral cover in *Acropora cervicornis*thickets: Potential relationships between density, growth, and polymorphisms

**DOI:** 10.1002/ece3.5035

**Published:** 2019-03-29

**Authors:** Crawford Drury, Justin B. Greer, Iliana Baums, Brooke Gintert, Diego Lirman

**Affiliations:** ^1^ Department of Marine Biology and Ecology, Rosenstiel School of Marine and Atmospheric Science University of Miami Miami Florida; ^2^ Department of Biology Pennsylvania State University University Park Pennsylvania; ^3^ Department of Marine Geoscience, Rosenstiel School of Marine and Atmospheric Science University of Miami Miami Florida

**Keywords:** *Acropora cervicornis*, clonality, density dependence, diversity, thicket

## Abstract

As coral reefs decline, cryptic sources of resistance and resilience to stress may be increasingly important for the persistence of these communities. Among these sources, inter‐ and intraspecific diversity remain understudied on coral reefs but extensively impact a variety of traits in other ecosystems. We use a combination of field and sequencing data at two sites in Florida and two in the Dominican Republic to examine clonal diversity and genetic differentiation of high‐ and low‐density aggregations of the threatened coral *Acropora cervicornis*in the Caribbean. We find that high‐density aggregations called thickets are composed of up to 30 genotypes at a single site, but 47% of genotypes are also found as isolated, discrete colonies outside these aggregations. Genet–ramet ratios are comparable for thickets (0.636) and isolated colonies after rarefaction (0.569), suggesting the composition of each aggregation is not substantially different and highlighting interactions between colonies as a potential influence on structure. There are no differences in growth rate, but a significant positive correlation between genotypic diversity and coral cover, which may be due to the influence of interactions between colonies on survivorship or fragment retention during asexual reproduction. Many polymorphisms distinguish isolated colonies from thickets despite the shared genotypes found here, including putative nonsynonymous mutations that change amino acid sequence in 25 loci. These results highlight intraspecific diversity as a density‐dependent factor that may impact traits important for the structure and function of coral reefs.

## 1 INTRODUCTION

Coral reefs worldwide are threatened by a combination of local and global stressors, including anthropogenic climate change (Hoegh‐Guldberg et al., 2007). As contemporary coral reefs face increasingly stressful environments, the ability to respond to new conditions is critical for the persistence of individual species and the ecosystems they create. The potential for acclimation and adaptation in corals has been documented in increasing detail in recent years (Barshis et al., 2013; Bay & Palumbi, 2015; Bay, Rose, Logan, & Palumbi, 2017; Matz, Treml, Aglyamova, & Bay, 2018; Palumbi, Barshis, Traylor‐Knowles, & Bay, 2014), but factors such as diversity, genotype interactions, and density dependence of disturbance response in foundational species may also play a role in the short‐term sustainability of some ecosystems (Reusch, Ehlers, Hammerli, & Worm, 2005).

The contemporary distribution of the branching stony coral *Acropora cervicornis*includes discrete, isolated colonies and very dense interlocking assemblages called thickets that may cover extensive substrate (Dustan & Halas, 1987; Goreau, 1959; Lirman et al., 2010; Morelock & Koenig, 1967). *Acropora cervicornis* thickets have traditionally been assumed to be monoclonal (Vargas‐Angel, Thomas, & Hoke, 2003) based on the high frequency of fragmentation (Tunnicliffe, 1981). Early observations using self‐recognition assays documented clonality among neighbors and, occasionally, colonies separated by tens of meters of substrate (Neigel & Avise, 1983). Genetic markers also document clonality of massive morphologies, suggesting that fragmentation is common for multiple species on Caribbean reefs (Foster, Baums, & Mumby, 2007; Foster et al., 2013; Manzello et al., 2018; Studivan & Voss, 2018). However, previous work shows that genetic diversity in *A. cervicornis*is present and variable over small spatial scales such as individual reefs, meaning that sexual reproduction and recruitment are also important drivers of contemporary populations (Drury et al., 2016; Reyes & Schizas, 2010; Vollmer & Palumbi, 2007). The congeneric *Acropora palmata* also occurs in both monoclonal and genotypically diverse assemblages that vary between individual reefs (Baums, Devlin‐Durante, & LaJeunesse, 2014; Baums, Miller, & Hellberg, 2006).

The density of thickets and sessile nature of corals creates the opportunity for ongoing interactions between individuals, which influence community response in other ecosystems (Hughes, Inouye, Johnson, Underwood, & Vellend, 2008; Stachowicz, Kamel, Hughes, & Grosberg, 2013). Because *A. cervicornis* populations on a reef can be diverse, dense aggregations with interlocking branches create potential for interactions between multiple genotypic combinations, but colonies that are not in close proximity may be less likely to be influenced by neighbors. High‐density negatively influences growth, branching rate, and survival in experimentally manipulated *A. cervicornis* (Griffin et al., 2015; Ladd, Shantz, Nedimyer, & Burkepile, 2016), but a notable gap exists at the intersection of density and diversity, including field or genetic research on corals naturally occurring at high densities.

Intra‐ and interspecific diversity influence various ecological outcomes in other ecosystems, including the structure of associated invertebrate communities (Johnson, Lajeunesse, & Agrawal, 2006), disease resistance (Zhu et al., 2000), nutrient cycling, stress resistance (Hughes & Stachowicz, 2004), resilience after disturbance (Reusch et al., 2005), and productivity (Huang et al., 2018). These impacts may be particularly pronounced in foundational species, which form structure that influences other species in the community (Barbour et al., 2009). Recent work has also documented interspecific diversity as an important factor in coral growth, survivorship, and productivity (Clements & Hay, 2019; McWilliam, Chase, & Hoogenboom, 2018), but our understanding of the potential influences of genotypic diversity or genet–genet interactions on community function in marine ecosystems is limited (Stachowicz, Bruno, & Duffy, 2007).

We use field and next‐generation sequencing data from isolated and thicket communities of the threatened coral *Acropora cervicornis* to examine (a) the genetic composition of each reef surveyed in Florida and the Dominican Republic, (b) differences in cover, growth, and bleaching impacts related to colony density, and (c) genetic differences between thickets and isolated colonies that may contribute to phenotype.

## 2 METHODS

### 2.1 Study sites

Sites with high density of coral were selected based on previous work and personal observation (Drury, Manzello, & Lirman, 2017; Lirman et al., 2010). Sunny Isles (~4 m depth) is found on nearshore consolidated hardbottom north of the Port of Miami in the northernmost region of the Florida Reef Tract, Cheetos (~3 m) is located in the central part of large patch reef north of Key Largo, approximately 75 km south of Sunny Isles. Cayo Carenero is near the mouth of Samana Bay in the Dominican Republic on a nearshore reef (~5 m), while Punta Rusia is on the exposed northern coast of the Dominican Republic (~7 m) detailed in Lirman et al. (2010). Among these sites, the colony morphology at Punta Rusia is unique, formed by extremely long branches and sparsely branching, open colonies. The Dominican Republic sites are approximately 235 km apart.

### 2.2 Sample collection

**Figure 1:**
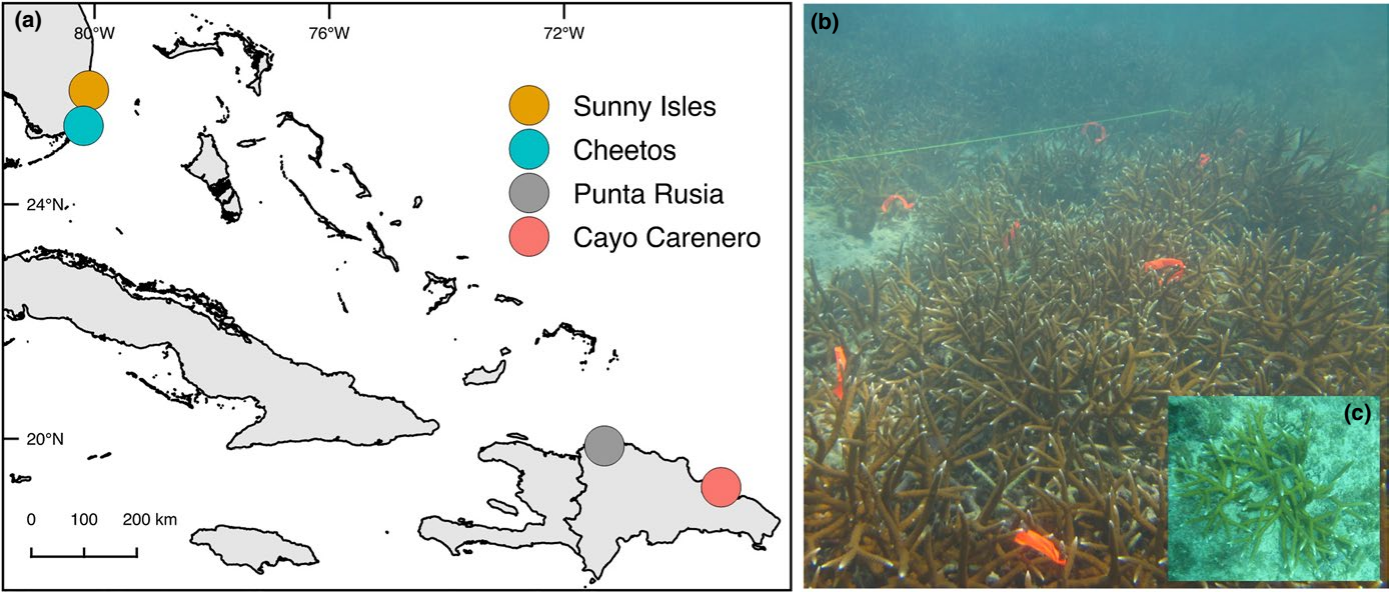
Map of sampling locations. (a) Two sites in the Dominican Republic and two sites in Florida were sampled, with collections from thickets and isolated colonies at each site. (b) Representative photographs of thicket from Sunny Isles composed of continuous interlocking skeleton at high densities. Orange flagging tape (foreground) was used to mark branches on a 1‐m grid for sampling. Line (background) marked the boundaries of the plots. (c) Photograph of representative isolated colony from Sunny Isles. Isolated colonies were collected >4 m from the boundaries of a thicket and were discrete units clearly originating from a single individual. Colony diameter is approximately 25 cm

Collections were made at two reef sites in Florida and two sites in the Dominican Republic (Figure 1a) between June 2014 and May 2015. At each site, samples were collected from (a) gridded plots covering continuous coral (Figure 1b) and (b) discrete, isolated samples from colonies outside the boundaries of the thicket (Figure 1c). Plots were randomly placed over areas of high coral cover and sized based on coral cover present to capture the largest continuous extent possible within a site (see Figure 1b).

Within thickets, a square was created with masonry line, and leaded line marked at 1 m intervals was moved in 1 m steps across the plot to create a grid. At each interval, flagging tape was used to mark the branch tip closest to the sampling point (Figure 1b) and a single branch tip (0.5 cm) was sampled. Rarely, areas of the thicket with no live coral cover required a branch to be selected up to ~20 cm from the marked point. For isolated collections, discrete colonies (originating from a single basal attachment or clearly continuous tissue of a single colony) at the same site >4 m from the boundary of the dense assemblage were haphazardly selected and a single sample was collected. Sampling colonies outside the thicket was limited by the availability of corals that met the spacing and isolation requirements. At Punta Rusia (Dominican Republic), isolated colonies were not present, so isolated colonies from a reef ~10 km away were sampled. In total, 100 samples were collected from each thicket in Florida, and 150 (Cayo Carenero) and 50 (Punta Rusia) were collected from each site in the Dominican Republic. The *A. cervicornis*assemblage in Punta Rusia was smaller than other sites, so sampling effort was focused on Cayo Carenero, causing the imbalance in sample size. In addition, 20–25 isolated colonies were sampled at each site.

Additional samples were collected from floating structures in the Miami‐Dade nursery (Drury, Schopmeyer, et al., 2017) to create intraindividual genetic differentiation thresholds for calling clones. Between 6 and 9 biological replicates from different branches of each of five genets were collected (*n* = 41 total). These biological replicates are “ramets” (i.e., pieces) of the same “genet” (i.e., genetically unique coral colony) and are expected to produce genetic distance patterns similar to asexual propagation of a genet across a wild reef.

All samples were collected with a clean razorblade by fragmenting 0.5 cm of each branch tip, selecting the apical only to minimize symbiont contamination. Samples were transferred to 250 μl of chaotropic salt preservative (4.5 M guanIdinium thiocyanate, 2% N‐laurylsarcosine, 50 mM EDTA, 25 mM Tris‐HCL pH 7.5, 0.2% antifoam, 0.1 M b‐mercaptoethanol). Samples were stored at 4°C until processing.

### 2.3 Photographs and landscape mosaics

To document the status of thicket assemblages and measure coral cover, photographs were collected at all sites. For the photograph surveys, a down‐facing dual still camera platform with two Nikon DSLR cameras was swum by a diver over the plot, collecting imagery at a rate of 1 image per second. Photographs were recorded approximately 2 m above the substrate at each site. To calculate percent cover for each plot at all sites, 10 raw images were randomly selected from each plot and 25 random points were assessed within each photograph using Coral Point Count (Kohler & Gill, 2006).

For the sites in Florida, images were assembled into a composite landscape mosaic following Gracias, Van Der Zwaan, Bernardino, and Santos‐Victor (2003) creating a single, spatially explicit image by combining many smaller overlapping images, each covering a small portion of the mapped seabed (Lirman et al., 2007).

### 2.4 Growth and bleaching data

Growth was measured within thickets and isolated colonies at both Florida sites during 2014–2015. No growth data were collected from the Dominican Republic due to logistical constraints. Cable ties were attached to sample branches of isolated colonies and colonies within thickets at 2 cm from the apical and then measured after ~6 months to compare growth rates of each assemblage. Rates were standardized to 1 year for comparison.

The thermal maximum in 2015 caused severe bleaching throughout the Florida Reef Tract (NOAA Coral Reef Watch 2015), so disturbance response was assessed at Cheetos Reef during this event. No data were available from the other three sites due to logistical constraints. Bleaching data were quantified through photographs of Cheetos thicket, a nearby outplant site (~30 m away) from Drury, Manzello, et al. (2017) and data from the Florida Reef Resilience Program (FRRP; http://frrp.org/). In 2015, only 92 *A. cervicornis*colonies were observed in FRRP surveys, equaling 0.038 colonies/m^2^ surveyed, which we consider to be highly isolated. Data from FRRP detail bleaching status by colony from two nonoverlapping 10 m × 1 m belt transect surveys at random reef sites throughout the Florida Reef Tract. We extracted bleaching status scores of all *A. cervicornis*surveyed in Miami‐Dade County and the Upper Florida Keys between September and October 2015. Outplanted corals compared within this site were outplanted in March 2015, 8 months prior to bleaching observations, and included 12 genets, including three local genets collected from within ~20 m of the outplant plot (Drury, Manzello, et al., 2017). There was no genotypic overlap between studies. Landscape photographs of Cheetos thicket were collected during September 2015. Random points (*n* = 46) were overlaid on these photographs, and bleaching status (Bleached, Partially Bleached, Pale, None) was assessed for each point. The same criteria were assessed in photographs of 52 outplanted corals on the same reef from August 2015 (Drury, Manzello, et al., 2017).

### 2.5 DNA isolation and library preparation

Corals from thickets (*n* = 319), isolated colonies (*n* = 75), and the nursery structures (*n* = 41) were processed. Samples consisting of skeleton and tissue were homogenized using silica beads in original collection tubes and extracted using a modified silica column and vacuum manifold protocol following Ivanova, Dewaard, and Hebert (2006). Samples were randomized at the DNA extraction step to minimize subsequent library preparation and sequencing artifacts. Each extracted sample was quantified (AccuBlue^TM^ High‐Sensitivity dsDNA Quantitative Solution), and 100 ng of DNA from each sample was dried down and rehydrated in 5 μl water. Libraries were prepared as in Drury et al. (2016) using a modified protocol of Elshire et al. (2011). Briefly, each library was digested with ApeKI to produce restriction fragments, which were bead‐purified to remove fragments <100 bp. 4–9 bp barcodes unique to each sample and a common adapter were ligated to fragments (see Elshire et al., 2011 for adapter sequences), and ligated samples were pooled and bead‐purified to select fragments in the 100–250 bp range. Pooled samples were PCR‐amplified for 18, 20, 22, and 24 cycles (Drury et al., 2016) using primers complementary to the oligonucleotides used in llumina flow cells to facilitate sequencing. PCR products were bead‐purified, eluted in 10 mM Tris, and analyzed via gel electrophoresis. All PCR products were run separately on an Agilent Bioanalyzer, and the library with the highest concentration of fragments from 200 to 300 bp was selected. Samples were sequenced as part of a larger project across three lanes using single‐end 75 bp reads on an Illumina HiSeq 2500 (Elim Biopharmaceuticals Inc., Hayward, CA).

### 2.6 Sequence data processing

Raw sequences were processed using a parsing script modified from Melo, Bartaula, and Hale (2016) to remove reads without a barcode and cut site. Trimmomatic 0.32 (Bolger, Lohse, & Usadel, 2014) was used to remove low‐quality bases at the leading and trailing end of reads and to remove reads where a 4‐bp sliding window average read quality fell below a phred score of 20 as an initial filtration step. Reads were demultiplexed to sample according to barcode using a modified script from Melo et al. (2016). Reads (599,736 ± 819,421 [mean ±*SD*] per sample) were aligned to the *Acropora digitifera* genome v1.1 to exclude symbiont reads and improve alignment (Shinzato et al., 2011) using Bowtie2 with default settings (Langmead, Trapnell, Pop, & Salzberg, 2009). Alignment files were analyzed using ANGSD (Korneliussen, Albrechtsen, & Nielsen, 2014), which incorporates genotype uncertainty into analyses and is therefore useful for low read depth data.

To examine clonality, samples from each site were analyzed alongside the biological replicates (i.e., branches from the same genet). Next, the identity‐by‐state (IBS) function was used with a randomly sampled base at each site with quality score >25 and mapq >30 to produce a distance matrix following Manzello et al. (2018). This strategy reduces the bias due to low/variable number of reads by randomly sampling bases instead of calling variants. Natural fragmentation would be expected to produce patterns of genetic distance between branch replicates (i.e., ramets) of the same genet similar to those found between ramets of the same genet on a wild reef, so replicates were used to create a threshold for assessing the contributions of fragmentation in the larger dataset.

To examine loci which vary between thickets and isolated colonies, data from each group were pooled across sites and then analyzed separately for thickets and isolated colonies. This sampling did not include any outplanted corals or nursery collected corals. Genotype likelihoods were calculated for each sample using the GATK method implemented in ANGSD, then major and minor alleles were inferred from these likelihoods and polymorphism was assessed on a per site basis with a *p*‐value cutoff of 2 × 10^−6^. These inputs were used with the expected‐maximization algorithm (Kim et al., 2011) to produce allele frequencies for each locus in thickets and isolated colonies. Frequencies were compared between assemblages by classifying each locus as (A) matched (same or reversed major and minor allele) or (B) mismatched (any combination of different alleles). For matching alleles, the difference in allele frequency between thickets and isolated colonies was calculated and plotted. Frequency difference ~0.3 was used as a cutoff and all matched loci with an allele frequency difference larger than this cutoff (*n* = 129) and all loci with mismatched alleles (*n* = 169) (were retained for downstream processing (*n* = 298 loci called in 37 ± 35 mean ± 1*SD* samples).

We further tested that large allele frequency differences observed were not due to chance/demographics for the 298 loci examined downstream. Each sample was randomly re‐assigned to isolated or thicket, maintaining the original sample size, and the allele frequency differences were recalculated as above. This process was repeated 20 times, and a distribution of the allele frequency differences was created for each locus. A *z*‐score was calculated for the observed allele frequency differences between thickets and isolated groups for each locus in the random distribution, and the *p*‐value was calculated.

Match rate was recalculated for the randomly assigned groups, and the proportion of replicates which agreed with the original analysis was calculated. Low values for this proportion indicate that the mismatched alleles in the thicket and isolated comparison are unlikely due to chance.

### 2.7 Nonsynonymous SNP identification

For both matched and mismatched loci, genes and exons were identified using the *Acropora digitifera* transcript annotation from NCBI (ref_Adig_1.1_scaffolds.gff3.gz). A full list of genomic features containing each SNP can be found in Supporting Information Data S2, see Dryad. SNPs contained within exons of protein‐coding mRNAs were used for downstream analysis. For these loci, mRNA accessions from the transcript annotation were used to obtain FASTA sequences with NCBI Entrez (https://www.ncbi.nlm.nih.gov/sites/batchentrez). A custom script was then used to replace the appropriate base pair with the identified alternate nucleotide. mRNA sequences were aligned using EBI Omega (Sievers et al., 2011) to confirm proper base pair replacement. If a SNP was contained in more than one mRNA splice variant, each variant was assessed independently.

To examine whether SNPs with mismatched alternate alleles resulted in a change in protein, modified mRNA sequences were translated into protein sequences using Expasy translate (Gasteiger et al., 2003) and aligned using EBI Omega. Synonymous SNPs were further investigated to determine whether they were in an untranslated region (UTR) or in the coding region but were synonymous. Possible annotations for nonsynonymous SNPs without protein names were identified using NCBI blastp, hidden markov model protein domain searches with hmmer (v3.1b2), and transmembrane region identification with tmHMM (v2.0c).

### 2.8 Analyses

**Figure 2:**
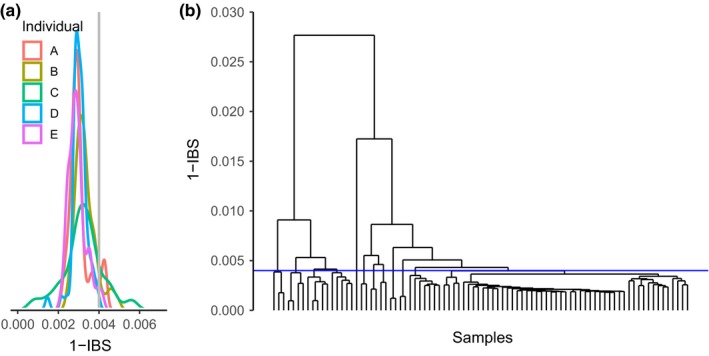
Between‐ramet pairwise genetic differences and genet trees. (a) Sequence data from biological replicates of five individual genotypes were used to create an identity‐by‐state (IBS) threshold for examining clonality. The 95th percentile of the distribution was then used in (b) hierarchical clustering analysis to describe clones from thickets and isolated colony samples within each site. Example data provided from Cheetos, all other clustering trees are in Supporting Information Figure S1

We modified the clonality analysis of Manzello et al. (2018), using the 95th percentile of identity‐by‐state (IBS) values for branch replicates of the same genet as a threshold (Figure 2a). The average pairwise genetic distance between branch replicates of the same genet was 0.0031 ± 0.0005 (Mean ± 1 *SD*) with a 95th percentile of 0.0041 (Figure 2a). This technique produces a more accurate clustering than a random hierarchical tree, but does not fully resolve each sample into a “monophyletic” group, so differences below this threshold are interpreted as a mixture of random sampling during IBS generation, somatic mutations, sequencing, and/or processing errors. Samples with pairwise values below this value were considered to be ramets of the same genet, and samples above this threshold were considered separate genets. To evaluate sitewide patterns in fragmentation, hierarchical clustering using the “complete” method was performed in R using *hclust*. This method creates similar clusters, avoiding the “chaining” of single‐linkage methods and produces trees where all samples within a node have a pairwise distance value lower than that node, effectively identifying a genet. The complete method favors identifying clusters where all pairwise differences are below a given value at the expense of potentially placing some ramets in separate clusters. Genet lists were created using *hclust* and plotted based on grid coordinates from sampled thickets. Genet lists were used to compare sharing of genets between thickets and isolated colonies, calculate distance between ramets within plots, and calculate diversity statistics including genet–ramet ratio (number of observed genets/number of samples: *N*_g_/*N*). These data were used with genets representing biological groups to calculate Shannon's diversity index and rarefied genet richness in the R package *vegan*(Oksanen et al.., 2010). Rarefaction clonal richness and *N*_g_/*N* were calculated for *n* = 14, the smallest sample size in any plot/isolated colony grouping.

To examine functional differences between thickets, a *t*‐test was used to compare growth between assemblages within sites for Cheetos and Sunny Isles. A two‐way chi‐square goodness‐of‐fit test was used to evaluate differences in distribution of bleaching status between FRRP data, outplanted corals, and thicket corals. A linear mixed model with random slope for Cover ~ Genet Diversity with site and plot as random effects was fitted using the R package *lme4* (Bates, Mächler, Bolker, & Walker, 2014). This model was compared to the null model with Genet Diversity removed with a likelihood ratio test for significance. Proportion of variance explained was calculated with the R package *MuMIn*.

## 3 RESULTS

### 3.1 Genotypic diversity patterns

**Figure 3:**
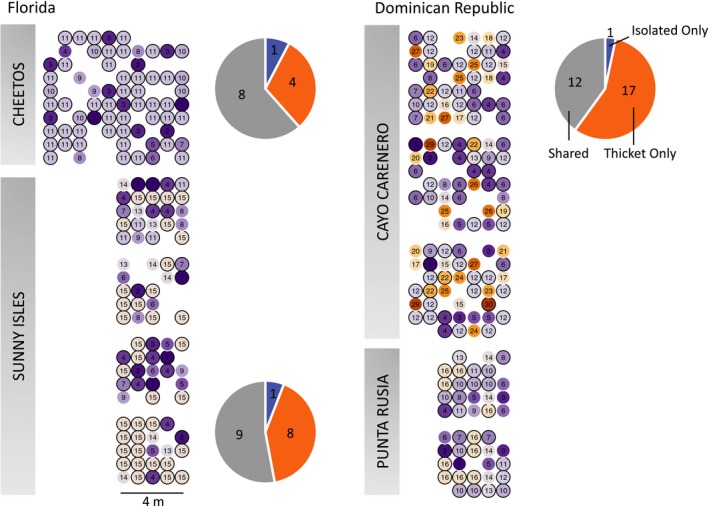
Spatial patterns of relatedness in thickets. Grids represent explicit spatial relationship of clonality as determined by hierarchical clustering. Plots are organized by site, with color and number corresponding to clonal identification of each sampling point. Similar colors and closer numbers represent shorter IBS distances. Clonal identification colors and numbers apply between plots within a site, but not across sites. Sampling points with a black border represent clones that were also found in the isolated colonies. Pie charts represent the proportion of clones that were found in either assemblage or shared on a sitewide basis, with the number of genets on each slice. There were no isolated colonies at the same site for Punta Rusia, so colonies were collected ~10 km away, and shared genets are not calculated. Gaps represent samples that were excluded due to sequencing issues. Scale bar relative to within‐plot samples, not between plots

**Figure 4:**
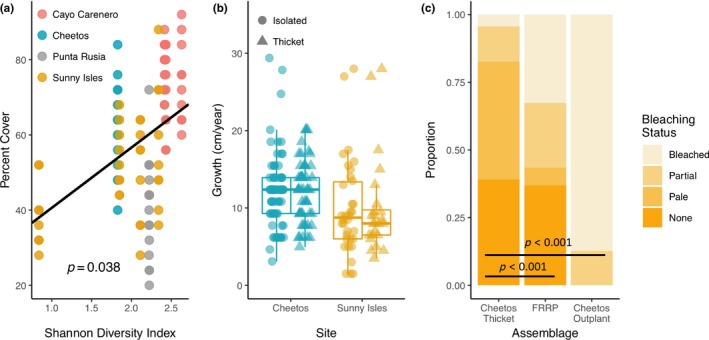
Diversity and site influence on cover, growth, and bleaching. (a) The best fit from the linear mixed model between percent cover of a plot and its diversity, determined by treating clonal groups as “species” within Shannon's Diversity Index calculations. Colors represent sites as in Figure 1a. Two separate plots from Punta Rusia are present, but the Diversity index was almost identical so they are difficult to distinguish. (b) Growth distributions of isolated (circles) and thicket (triangles) colonies at Cheetos and Sunny Isles with colors representing sites as in (a). (c) Bleaching status as assessed by Florida Reef Resilience Program (FRRP) for Cheetos thicket corals, outplanted corals, and FRRP‐surveyed individuals in Miami‐Dade and Broward counties. Significance values are for chi‐square tests for all comparisons and for the comparison of only FRRP and thicket

Using the 95th percentile as the between genet cutoff for identity‐by‐state, there were between 13 and 30 genets per site (Figure 2b, Supporting Information Figure S1), with a generally higher genet–ramet ratio (*N*_g_/*N*) found in isolated colonies than in thickets at each site before and after rarefaction (Table 1). Thickets and isolated colonies share genets at each site, with between 40% and 60% of genets found in both assemblage types (Figure 3). Few genets are exclusively found as isolated colonies. Ramets of the same genet were found near the maximum distance possible given the plot design, and genet maps show that ramets were spread over 20 m at Sunny Isles (Figure 3). *N*_g_/*N* also varied extensively within a site. *N*_g_/*N* is higher in isolated colonies than thicket plots, falling between 0.450 and 0.714 at Cheetos and Cayo Carenero. Overall, Punta Rusia (DR) was the most genotypically diverse site with the highest sitewide *N*_g_/*N*, while Cheetos (FL) was the least diverse (Table 1). In general, the Dominican Republic was more genotypically rich, with higher average Shannon's index and rarefied clonal richness values compared to Florida. Cheetos contains a notable outgroup with five samples that are substantially different from others at the site. Sunny Isles contains a very large genotype that appears to be extensively fragmented (Supporting Information Figure S1). Percent cover is significantly related to Shannon's Diversity Index across all sites and plots (likelihood ratio test: ($\chi_{\left(1\right)}^{2}$ = 4.28, *p* = 0.038), Figure 4a). The fixed and random effects in this model explain 55.3% of the variance.

**Table 1:** Diversity and sampling statistics for each site

Site	Assemblage	*N*	Genet	*N*_g_/*N*	Shannon Diversity Index (*H*′)	Rarefied clonal richness	Rarefied * N*_g_/*N*	Average distance	Max distance
Cheetos	ALL	92	13	0.141	—	—	—	—	—
	ISO	20	9	0.450	1.843	7.1	0.509	—	—
	P1	72	12	0.167	1.827	6.1	0.437	5.2	12
Cayo Carenero	ALL	136	30	0.221	—	—	—	—	—
	ISO	21	13	0.619	2.310	9.5	0.681	—	—
	P1	41	17	0.415	2.415	8.6	0.617	3.6	8.5
	P2	35	18	0.514	2.630	9.8	0.702	3.6	7.2
	P3	39	18	0.462	2.432	9.0	0.642	3.7	6.7
Punta Rusia	ALL	64	16	0.250	—	—	—	—	—
	ISO	20	11	0.550	2.221	8.9	0.639	—	—
	P1	22	11	0.500	2.221	8.7	0.620	2.3	4.2
	P2	22	12	0.545	2.224	8.8	0.631	2.3	4.2
Sunny Isles	ALL	102	17	0.167	—	—	—	—	—
	ISO	14	10	0.714	2.206	10.0	0.714	—	—
	P1	22	5	0.227	0.839	3.8	0.271	2.6	5.7
	P2	21	10	0.476	2.112	8.2	0.589	2.6	4.5
	P3	19	9	0.474	1.850	7.5	0.536	2.7	5
	P4	26	14	0.538	2.341	9.1	0.647	2.7	4.5

Note

Genets are number of clones as determined by hierarchical clustering, *N*_g_/*N* is the Genet–Ramet ratio, Simpson and Shannon are diversity indices calculated by treating clonal groups as “species,” and rarefied clonal richness was corrected for the minimum number of samples (*n* = 14, Sunny Isles isolated colonies) and divided by minimum number (*n* = 14) to calculated rarefied *N*_g_/*N*.

### 3.2 Growth and disturbance response

Growth was not significantly different between thicket colonies and isolated colonies at Cheetos (*t*_(122)_ = 0.032, *p* = 0.975) or Sunny Isles (*t*_(56)_ = 0.006, *p* = 0.995; Figure 4b). Only 4% of Cheetos thicket corals were fully bleached, compared to 87% of outplanted colonies at the same reef and 32% of regional FRRP‐surveyed colonies (Figure 4c). While some corals remained “healthy” in the thicket and FRRP surveys, outplanted corals all showed partial bleaching or paling. The distribution of bleaching status between datasets is significantly different ($\chi_{\left(\text{6},\;\text{193}\right)}^{2}$ = 110.3, *p* < 0.001), and a direct comparison between thicket and FRRP data is also significantly different ($\chi_{\left(\text{3},\;\text{138}\right)}^{2}$ = 34.6, *p* < 0.001). The proportion of nonbleached colonies in thickets (39%) and FRRP data (36%) is approximately equal; however, the severity of bleaching in colonies that did suffer from stress is substantially higher in FRRP corals (Figure 4c).

### 3.3 Allelic differentiation

Of 30,985 loci investigated in this study, 87.5% had matching major and minor alleles for thickets and isolated colonies, 12.0% had a reversal of major and minor alleles, and 0.5% had a different major or minor allele. 298 loci were further investigated, including 129 loci with large differences (>0.3) in allele frequency and 169 loci with different alleles. Of these, 175 (57.8%) were within a gene and 79 (26.7%) were within exons in those genes, including 70 loci in protein‐coding regions of mRNAs, eight in long noncoding RNAs, and one for valine tRNA (Supporting Information Data S2, see Dryad). 89 loci (71% of those analyzed with large differences in allele frequencies) had significant *z*‐scores after Bonferroni correction (*p* < 0.0004). Among SNPs with different alleles in thicket and isolated colonies, 140 (83%) had a distinct pattern in observed changes when compared with random resampling (i.e., less than 20% of replicates were also mismatched). Nonsynonymous changes in nucleotides between groups resulting in different amino acid sequences were identified for 25 loci (Table 2).

**Table 2:** Table of nonsynonymous mutations with functional annotations from embl‐ebi quickGO

Dataset	Gene ID	RNA accession	RNA name	Function
Mismatched alleles	107354865	XM_015921357.1	CUB and sushi domain‐containing protein 3‐like	Regulation of dendrite development
	107354343	XM_015920805.1	Excitatory amino acid transporter 1‐like	High‐affinity glutamate transmembrane transporter activity, neurotransmitter transport
	107337607	XM_015902809.1	Leucine‐rich repeats and immunoglobulin‐like domains protein 1	Sensory perception of sound
	107336334	XM_015901416.1	Nucleotide‐binding oligomerization domain‐containing protein 1‐like	NF‐kappa‐B activity
	107356826	XM_015923450.1^a^	Pleckstrin homology domain‐containing family G member 5‐like	Signal transduction
	107327972	XM_015892693.1	Proteasome subunit beta type‐7‐like	Intracellular protein degradation
	107334554	XM_015899506.1	Sushi von Willebrand factor type A EGF and pentraxin domain‐containing protein 1‐like	Calcium ion binding
	107351227	XM_015917525.1	THAP domain‐containing protein 1‐like	Regulates endothelial cell proliferation
	107356265	XM_015922901.1	Trichohyalin‐like	Transition metal ion binding
	107329819	XM_015894489.1	Uncharacterized LOC107329819	
	107339633	XM_015904950.1	Uncharacterized LOC107339633	
	107339930	XM_015905281.1	Uncharacterized LOC107339930%2C transcript variant X1	
	107340735	XM_015906112.1^a^	Uncharacterized LOC107340735	
	107341044	XM_015906471.1^a^	Uncharacterized LOC107341044	
	107347468	XM_015913405.1	Uncharacterized LOC107347468	
	107348660	XM_015914711.1	Uncharacterized LOC107348660	
	107348864	XM_015914924.1	Uncharacterized LOC107348864	
	107350464	XM_015916691.1	Uncharacterized LOC107350464	
	107356306	XM_015922928.1^a^	Uncharacterized LOC107356306	
	107356511	XM_015923119.1	Uncharacterized LOC107356511	
Matched alleles	107349183	XM_015915289.1	C‐C chemokine receptor 1‐like protein 1%2C transcript variant X2	Signal transduction via increasing intracellular C^2+^
	107333182	XM_015897952.1	Phosphatidylinositol phosphatase PTPRQ‐like	Dephosphorylation of phosphatidylinositol phosphates
	107345218	XM_015910931.1	PiggyBac transposable element‐derived protein 4‐like	DNA binding
	107334333	XM_015899269.1	Protein LTV1 homolog	Production of 40S ribosomal subunit
	107358358	XM_015924967.1	Uncharacterized LOC107358358	

Notes

Dataset indicates loci from the mismatched pairs with different major or minor alleles or the matching dataset with large differences. See Supporting Information Table S1 for all loci in analysis. RNA names in italics for uncharacterized loci represent our annotations (see Section 2).

a Mutations that result in a premature stop codon.

## 4 DISCUSSION

We examine the threatened staghorn coral *Acropora cervicornis* at a range of contemporary natural densities in Florida and the Dominican Republic, finding differential clonal propagation and allelic differences between thickets and isolated colonies. We also found evidence of a positive relationship between coral cover and genotypic richness across sites and plots, which we believe is the first evidence for a functional outcome of genotypic diversity in reef‐building corals.

Clonality patterns found here show that both fragmentation and sexual recruitment contribute to the creation of thickets. The *N*_g_/*N* varied over threefold within thicket plots, representing nearly monoclonal and diverse assemblages influenced by sexual recruitment, respectively (Figure 3). Differences in this scale indicate that thickets can be structured by various mechanisms, such as genets growing together, single large genets propagating over local substrate through fragmentation (e.g., Sunny Isles Plot 4, Cheetos Plot) and through the influence of sexual recruitment. High clonality and genets shared between thickets and isolated colonies may arise from the transport of fragments from the thickets to the periphery as a result of physical disturbance or the dieback of previously continuous thickets, leaving some ramets isolated from the remainder of the genet. The variation between highly clonal and highly diverse plots at different sites highlights the spatial heterogeneity of sexual recruitment as an influence on thicket development.

Genet–ramet ratios described here are similar to the highest *N*_g_/*N* found in previous work on nonthicket samples (Irwin et al., 2017) and within the range documented for *A. palmata* throughout the Caribbean (Baums et al., 2014, 2006), although sampling efforts and design between studies were different and can influence these values. In isolated colonies, *N*_g_/*N* confirms that many colonies of *A. cervicornis* on a single reef are of sexual origin and confirms observations of diverse nonthicket assemblages of Acroporids on modern reefs (Drury et al., 2016; Reyes & Schizas, 2010). Differences in *N*_g_/*N* between thickets and isolated colonies may also be influenced by sampling design; the use of gridded plots to collect thicket corals is more likely to resolve clonality than haphazard collections of isolated colonies. To correct for this, we include rarefied *N*_g_/*N*, which show slightly higher values in isolated colonies but are surprisingly similar, highlighting the importance of density or interactions between colonies. Site‐specific patterns in genotypic diversity may also form a mosaic within larger regions, likely depending on environmental conditions, habitat availability, historical population dynamics (Baums et al., 2006), and disturbance history (Connell, 1997; Connell et al., 2004).

The highest coral cover is found in plots with the highest genotypic diversity, implying that more diverse coral assemblages facilitate higher coral cover. This outcome contrasts previous findings where ecological performance was not positively related to genotypic diversity in Acroporids (Baums et al., 2006; Ladd et al., 2016; Williams, Miller, & Baums, 2014); however, it is possible that a colony density threshold must be met to observe this relationship. If this is the case, this requirement would support the idea that interactions between colonies are playing a role in the development of thicket cover.

We were only able to measure growth rates at the two sites in Florida, where growth was similar between thickets and isolated colonies, suggesting that differences in coral cover are a result of mortality or disturbance response and not unusual patterns of growth within thickets. Likewise, we were only able to obtain bleaching data from a single site, where we attempted to compare various nonexperimental information on bleaching from this time frame using data from an outplanting experiment on the same reef (Drury, Manzello, et al., 2017) and concurrent regional bleaching data (FRRP). The hypothesis of differential disturbance response is supported by these data, although our analysis is opportunistic due to the natural thermal stress event. Thicket corals at Cheetos bleached less frequently and less severely than FRRP and outplanted corals, which may be related to density effects of the interlocking spatial arrangement.

Several factors limit the interpretation of bleaching comparisons. First, outplanted colonies were substantially smaller than the fully mature colonies comprising thicket and isolated communities, and size‐specific bleaching response could influence this outcome (Brandt, 2009). Second, our comparisons are limited to a single site and are confounded by the lack of definitive genotypic overlap between thicket corals, outplanted corals, and FRRP corals, so results could be influenced by genotypic response masked by thicket membership. We also did not have data on isolated colonies sequenced in this experiment. Nevertheless, 12 genets were outplanted at this site and suffered high bleaching stress, with at least 75% of ramets bleaching in all genets (Drury, Manzello, et al., 2017), including three different genets from that site. In addition, FRRP corals were sampled across 14 sites spanning ~60 km, likely capturing much of the natural variability in this region (Drury, Schopmeyer, et al., 2017). Although our data do not allow for direct comparisons of these trends, we think that a link between aggregation type and bleaching response is suggested by the data and warrants further investigation.

We also find genomic differences between corals occurring in thickets and isolated colonies, so allelic composition may be associated with density, structure, or function. Our statistical validation indicates these differences in allele frequency attributed to thickets or isolated colonies are infrequently due to chance or demographic (Supporting Information Data S2, see Dryad). Nonsynonymous mutations like those found here indicate polymorphisms that are the most likely to have direct impacts on function by altering secondary protein structure, particularly if the change is to a dissimilar amino acid in a comparison with different alleles. Several nonsynonymous SNPs were found in genes integral to cell survival and stress response. For example, a nonsynonymous SNP in pleckstrin homology family G member 5 results in a premature stop codon in isolated colonies and an amino acid change in the thicket colony sequences (Table 2). The shortened sequence is 222 amino acids, compared to 1,322 amino acids for the full protein, likely resulting in production of a nonfunctioning protein. Importantly, congeneric *Acropora palmata* exhibited differential expression of this gene due to thermal stress between genetically distinct larval families in Puerto Rico (Polato, Altman, & Baums, 2013), suggesting a functional role in *Acropora* species. Many of the genomic differences between thickets and isolated colonies have been implicated in previous research on thermal tolerance in Acroporids, including protein tyrosine kinase receptors, zinc finger proteins, ubiquitin, and ankyrin (Supporting Information Data S2, see Dryad; Barshis et al., 2013; Dixon et al., 2015; Palumbi et al., 2014; Polato et al., 2013; Rose, Seneca, & Palumbi, 2015).

Synonymous mutations may have fewer direct effects but can still alter function. Codon bias mutations to nonpreferred codons can significantly decrease protein production (Carlini & Stephan, 2003) and synonymous SNPs may also affect splicing, resulting in nonneutral changes (Pagani, Raponi, & Baralle, 2005). Synonymous mutations within protein‐coding exons were observed for several potentially important proteins, including multidrug resistance protein 4 and cytochrome P450. Multidrug resistance protein 4 is a versatile protein which removes substances from cells and may have a key function cell signaling (Russel, Koenderink, & Masereeuw, 2008) and cytochrome P450 3A8 (CYP3A8), involved in cnidarian chemical defense and stress response (Goldstone, 2008) and heat‐stress response of *Symbiodinium* (Rosic, Pernice, Dunn, Dove, & Hoegh‐Guldberg, 2010). While neither synonymous nor nonsynonymous mutations guarantee functional outcomes, these data suggest that some stress response SNPs may result in changes that alter protein expression or function, creating differences in stress response between isolated and thicket corals. It is important to note one limitation of this analysis is that pooling all thicket and isolated samples from across regions could reflect demographic differences. This may happen in cases where coverage across individuals from certain populations coincided with low coverage in others, but we chose to analyze all data together to increase the power of describing conserved differences based on assemblage type and not demographics.

Diversity outcomes (such as differences in coral cover) can be partitioned into selection and complementarity effects in controlled experiments, where either sampling or facilitation/niche partitioning influences the outcome (Loreau & Hector, 2001). Selection effects occur as the probability of including a genet with a specific trait (e.g., high growth rate in corals translating to high localized cover) increases as diversity increases (Stachowicz et al., 2007). Without explicit data on the performance of specific genets it is impossible to parse selection and complementarity in our results; however, some patterns may be informative. Calculating Shannon's Diversity Index rather than using number of genet accounts for both richness and evenness, while spatial maps highlight the presence of a range of naturally occurring genets, suggesting that selection of hyper‐successful individuals is unlikely to be driving differences in coral cover. Thus, the positive relationship between genetic diversity and coral cover could relate to complementarity, influencing cover through facilitation, niche partitioning or genet by genet interactions (Hughes et al., 2008). It is also possible that the reverse is true, where density facilitates diversity through some impact on sexual reproduction or recruitment. While we find potential to be less likely, our data do not allow us to conclusively parse cause and effect.

Experimental manipulation of genotypic diversity in *A. cervicornis* did not influence growth or partial mortality in previous work (Ladd et al., 2016), but the authors point out that nonrandom or artificial distribution of genets or limited diversity may have influenced this outcome. Experimental manipulation of species diversity in a restoration project was also inconclusive (Cabaitan, Yap, & Gomez, 2015), and a meta‐analysis found weak negative influence of diversity on resistance and resilience in coral reefs (Zhang et al., 2014). Recent work on Pacific species assemblages has documented increased productivity in multispecies treatments that is also dependent on surface area of colonies (McWilliam et al., 2018). Diversity can also increase growth and decrease tissue mortality in polyculture (Clements & Hay, 2019), but the impact of intraspecific diversity remains poorly understood.

Studies of diversity effects are typically confounded by density (Stachowicz et al., 2007), and we assume that coral cover is related to colony density in the present study. Coral density negatively impacts growth and branching in experimentally manipulated *A. cervicornis* (Griffin et al., 2015; Ladd et al., 2016), correlates with growth anomalies (Aeby et al., 2011), and influences associated invertebrate communities in Caribbean Acroporids (Baums, Miller, & Szmant, 2003a, 2003b). *Acropora* corals are the preferred but not the only prey of gastropod coral predators which leads to the aggregation of gastropods on coral recruits and remnants of previous thickets causing colony death (Baums, Miller, & Szmant, 2003b; Knowlton, Lang, & Keller, 1990). Density may be temporally variable in coral reef habitats dominated by *Acropora cervicornis* colonies, which can be transient, with high fragmentation rates and dynamic spatial patterns (Highsmith, 1982; Walker, Larson, Moulding, & Gilliam, 2012). In this context, coral genets can be extremely long‐lived, even if specific ramets are not (Devlin‐Durante, Miller, Precht, & Baums, 2016; Irwin et al., 2017). Structural complexity facilitates survivorship during asexual propagation via retention and stabilization of fragments, initiating and maintaining thickets in Acroporid corals (Baums et al., 2006). Coral density also interacts with habitat characteristics such as presence of solid substrate (Lirman, 2000) and reef slope (Baums et al., 2006; Foster et al., 2013) and the occurrence of physical disturbance (e.g., hurricanes) to influence the rate of successful fragmentation (Fong & Lirman, 1995; Hughes, 1994; Lirman, 2000). Reefs with low coral diversity may suffer from the allee effect, especially in an obligately outcrossing species such as *A. palmata* (Baums, Hughes, & Hellberg, 2005), although *A. cervicornis* may be somewhat more susceptible to self‐crossing (Fogarty, Vollmer, & Levitan, 2012). Conversely, high density of diverse genets may facilitate local sexual reproduction in an “anti‐allee” effect (Knowlton, 1992).

Our study sites have much higher coral cover than previous work in *A. palmata*, where natural plots contained 2%–5% live coral (Williams et al., 2014; Williams, Miller, & Kramer, 2008) and experimental plots of *A. cervicornis*, which contained 10%–20% cover (J. Griffin & M. Ladd, personal communication). We hypothesize that a density threshold exists, such that corals growing within a tight framework such as an *A. cervicornis* thicket may facilitate nonadditive effects leading to increased coral cover and potential differences in stress response. Under this hypothesis, structural complexity maintains higher survivorship during asexual propagation via physical capture of complex fragments which bind to the substrate in place, reducing mortality and further enhancing overall diversity. This process could be especially important if it creates a measurable difference in survivorship in *A. cervicornis*, which can be locally ephemeral. Although high‐density assemblages influence growth in experimental settings, specific genet interactions or local adaptation could counteract this pattern in natural systems. Thermal stress response could also be a developed characteristic of higher density in thickets via self‐shading (Goreau & Macfarlane, 1990) or possible disruption of flow characteristics in and around colonies (Nakamura & Van Woesik, 2001), leading to small but potentially important differences in bleaching severity or occurrence. Further, thickets reduce predation pressure on individual ramets via a dilution effect (Baums et al., 2003b). This hypothesis reflects patterns of complementarity, where niche partitioning or interactions among individuals that only develop in dense assemblages produce effects absent or impossible among more sparsely populated reefs.

As coral reefs continue to face challenging conditions, resilience or resistance is valuable in any form. Here, we show genetic diversity and high‐density assemblages such as thickets of the threatened coral *A. cervicornis*may help increase coral cover and impact bleaching stress, providing additional time for evolutionary processes to allow corals to adapt to stressful conditions.

## CONFLICT OF INTEREST

None declared.

## AUTHOR CONTRIBUTIONS

CD and DL designed the study. CD, DL, and BG collected data. CD and JG analyzed the data. CD, JG, DL, IB, and BG wrote and edited the paper.

## Data Availability

Sequencing data available on NCBI SRA SUB5182953. Mosaic files are uploaded to Dryad https://doi.org/10.5061/dryad.s4rr2vf.
