# Supplementary figures and images for: Clonal diversity impacts coral cover in Acropora cervicornisthickets: Potential relationships between density, growth, and polymorphisms
Source: Ecol Evol. 2019 Mar 29;9(8):4518–31. doi: 10.1002/ece3.5035 (PMC6476746; doi:10.1002/ece3.5035)

Cayo Carenero

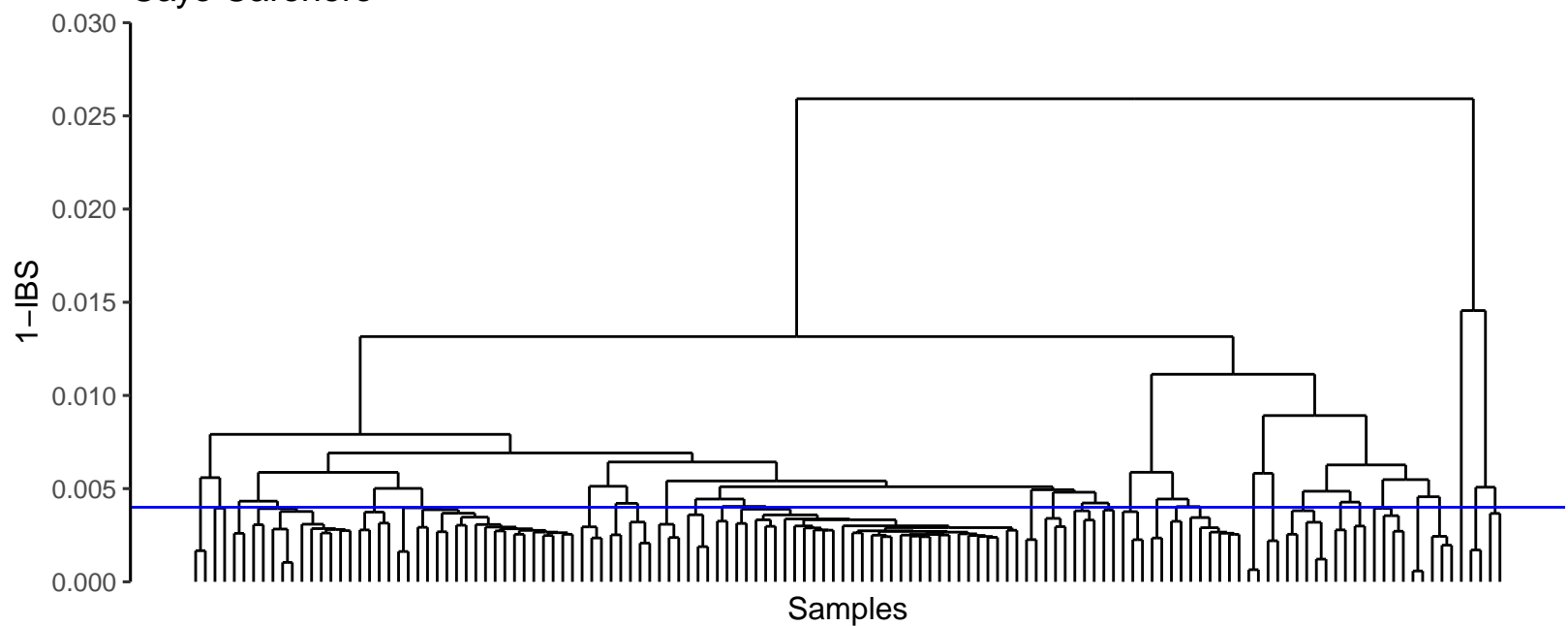

Punta Rusia

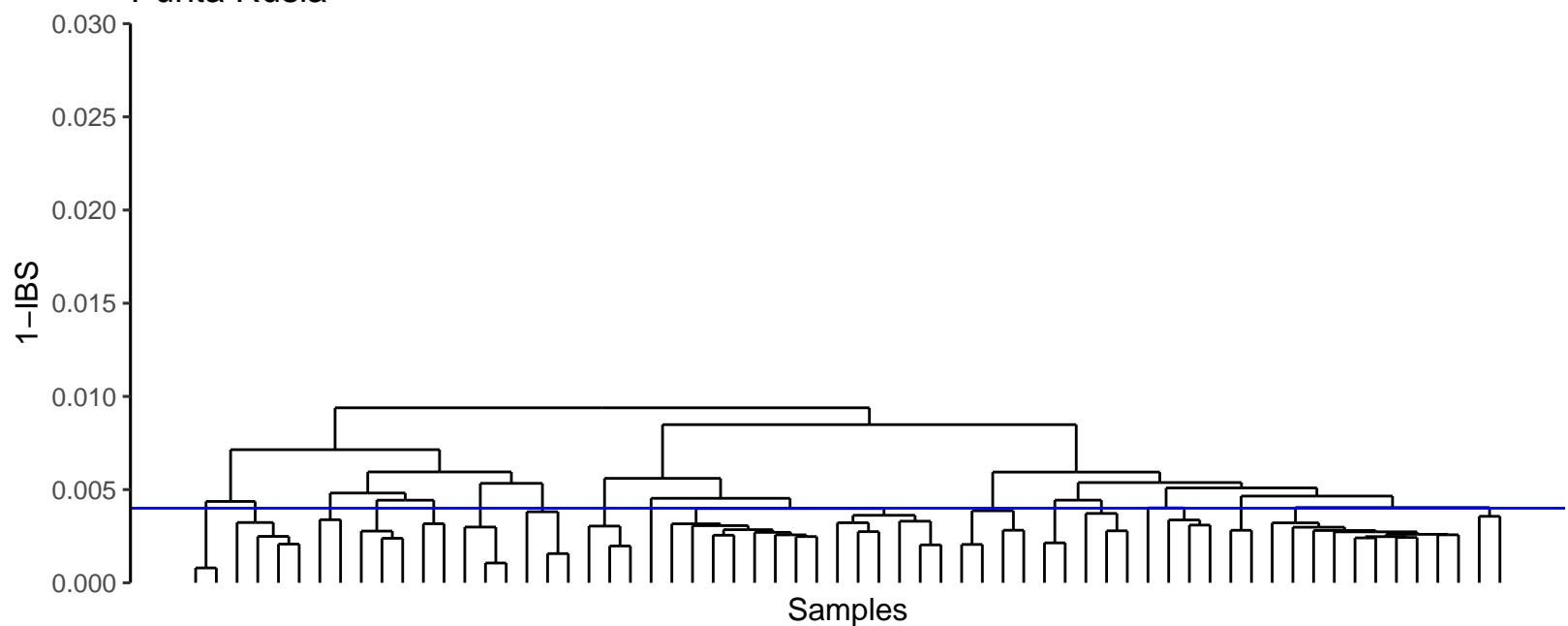

Sunny Isles

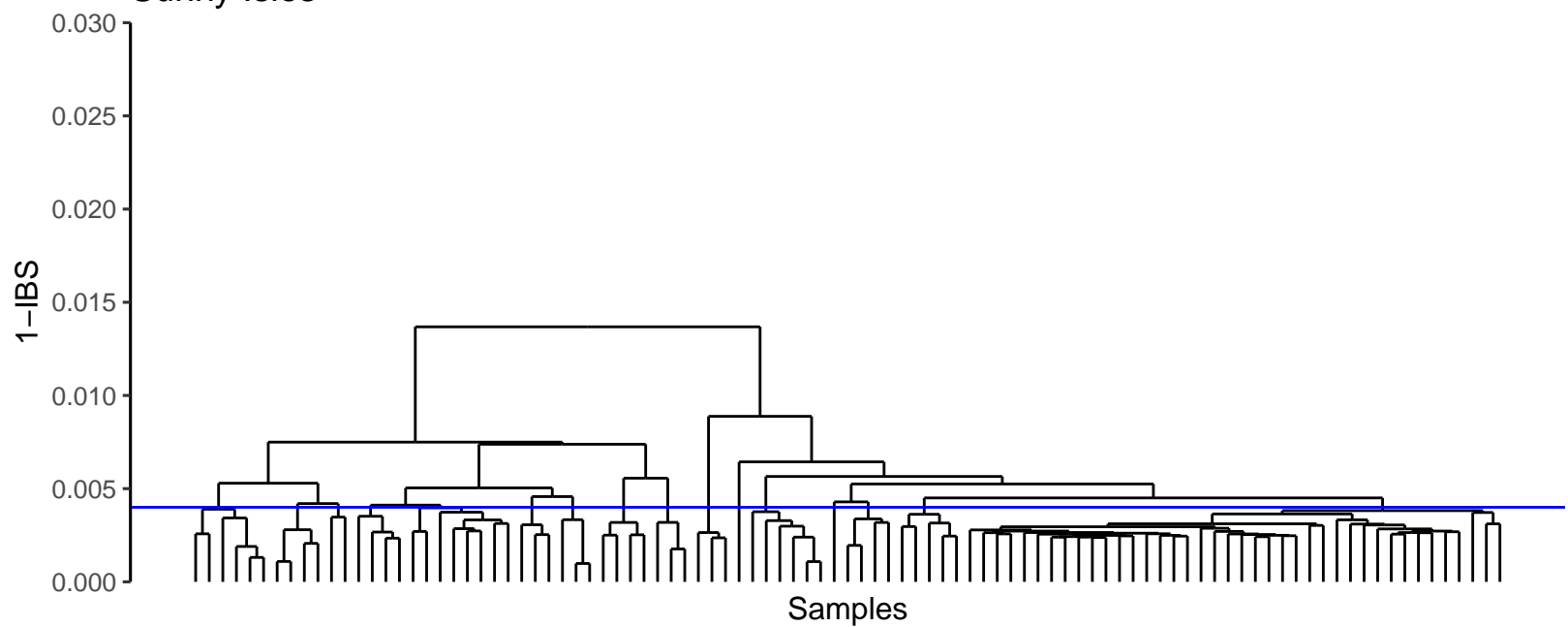

Supplement: Supplementary file 1 [file ECE3-9-4518-s001.pdf]
