# Supplementary material for: Clonal diversity impacts coral cover in Acropora cervicornisthickets: Potential relationships between density, growth, and polymorphisms
Source: Ecol Evol. 2019 Mar 29;9(8):4518–31. doi: 10.1002/ece3.5035 (PMC6476746; doi:10.1002/ece3.5035)
Supplement: Supplementary file 3 [file ECE3-9-4518-s003.docx]

*Supplemental Figure 1 - Within-ramet pairwise genetic differences and genet trees*

Hierarchical clustering analysis of each sample within a site, using the 95^th^ percentile of the distribution of IBS as a clonality cutoff (blue line) to describe clones from thickets and isolated colony samples within each site. An additional example can be found in Figure 2b.

*Supplemental Figures 2-6 – Mosaic images for Florida plots*

*Supplemental Table 1 – Annotations and Locus Specific analyses for randomization*

Data on randomization results by locus for the 298 loci analyzed between Thickets and Isolated colonies. Proteins for all polymorphisms found in exons, potential gene annotations.
